# Discovery of Genes Activated by the Mitochondrial Unfolded Protein Response (mtUPR) and Cognate Promoter Elements

**DOI:** 10.1371/journal.pone.0000874

**Published:** 2007-09-12

**Authors:** Jonathan E. Aldridge, Tomohisa Horibe, Nicholas J. Hoogenraad

**Affiliations:** Department of Biochemistry, La Trobe University, Melbourne, Victoria, Australia; Baylor College of Medicine, United States of America

## Abstract

In an accompanying paper, we show that the mitochondrial Unfolded Protein Response or mtUPR is initiated by the activation of transcription of *chop* through an AP-1 element in the *chop* promoter. Further, we show that the *c/ebpβ* gene is similarly activated and CHOP and C/EBPβ subsequently hetero-dimerise to activate transcription of mtUPR responsive genes. Here, we report the discovery of six additional mtUPR responsive genes. We found that these genes encoding mitochondrial proteases YME1L1 and MPPβ, import component Tim17A and enzymes NDUFB2, endonuclease G and thioredoxin 2, all contain a CHOP element in their promoters. In contrast, genes encoding mitochondrial proteins Afg3L2, Paraplegin, Lon and SAM 50, which do not have a CHOP element, were not up-regulated. Conversely, genes with CHOP elements encoding cytosolic proteins were not induced by the accumulation of unfolded proteins in mitochondria. These results indicate that mtUPR responsive genes appear to share a requirement for a CHOP element, but that this is not sufficient for the regulation of the mtUPR. A more detailed analysis of promoters of mtUPR responsive genes revealed at least two additional highly conserved, putative regulatory sites either side of the CHOP element, one a motif of 12 bp which lies 14 bp upstream of the CHOP site and another 9 bp element, 2 bp downstream of the CHOP site. Both of these additional elements are conserved in the promoters of 9 of the ten mtUPR responsive genes we have identified so far, the exception being the Cpn60/10 bidirectional promoter. Mutation of each of these elements substantially reduced the mtUPR responsiveness of the promoters suggesting that these elements coordinately regulate mtUPR.

## Introduction

Cells respond to a wide variety of stresses through the transcriptional activation of genes that harbor stress elements within their promoters. The heat shock element (HSE) is found in promoters of genes encoding proteins representative of all compartments [1], enabling cells to respond to global stresses by the increased synthesis of heat shock proteins and other molecular chaperones involved in repair.

On the other hand, cells can also respond to stresses that are specific to individual organelles. For example, the endoplasmic reticulum (ER) unfolded protein response (erUPR) [2], wherein a wide range of genes encoding proteins involved in the maintenance of ER function are up-regulated in species ranging from yeast to human [3]. In mammalian cells, erUPR has three signaling pathways, Ire1 [4], PERK [5], and ATF6 [6], wherein PERK plays a major role in ER stress-induced translational attenuation [4]. ATF6 is activated by proteolysis and binds in the presence of NF-Y directly to the *cis* –acting element (CCAAT-N_9_-CCACG) to induce ER stress-inducible proteins which include molecular chaperones such as the ER isoform of HSP70 (also known as BIP or GRP78), GRP94, GRP170, calreticulin, peptidyl-prolyl-cis-trans-isomerase (FKBP13), protein disulfide isomerase (PDI), and PDI superfamily proteins ERp72, ERp57, and ERp29 [reviewed in 7]. The ATF6 pathway also activates transcription of the *chop* gene encoding a bZIP transcription factor CHOP (C/EBP homology protein). CHOP expression is regulated by a number of transcriptional and translational mechanism [8] and it has recently been shown that the induction of CHOP by erUPR leads to the transcriptional activation of BIM, leading in turn to apoptosis [9].

The mitochondrial matrix also contains its own set of molecular chaperones involved in the folding of newly imported proteins, and also for the folding of some of the 13 polypeptides encoded by mtDNA [10]. We previously reported on the discovery of a mitochondrial unfolded protein response (mtUPR) in mammalian cells, in which the accumulation of unfolded protein within the mitochondrial matrix resulted in the transcriptional upregulation of nuclear genes encoding mitochondrial stress proteins such as chaperonin 60 (Cpn60), chaperonin 10 (Cpn10), mtDnaJ and ClpP, but not those encoding stress proteins of the endoplasmic reticulum (ER) or the cytosol [11], [12]. Moreover, analysis of the *cpn60/10* bidirectional promoter identified a CHOP element (GG/ATTGCA) as the mitochondrial stress response (mtUPR) element and CHOP, in association with C/EBPβ, was shown to regulate expression of mitochondrial stress genes in response to the accumulation of unfolded proteins in the matrix of mitochondria [12]. Further studies suggest that mtUPR is regulated via a 2 stage process, involving the transcriptional activation of a primary set of genes (*chop, c/ebpβ)* which subsequently activate transcription of mtUPR responsive genes containing the CHOP element [13].

In this report, we investigated whether other genes encoding mitochondrial proteins involved in quality control are also up-regulated by mtUPR. It was found that mtUPR responsive genes all have a CHOP element in their promoters, whereas genes encoding mitochondrial proteins, which do not have the CHOP element, were not up-regulated. These results indicate that mtUPR activates genes through a CHOP dependent pathway. Bioinformatics analysis of ten mtUPR responsive genes shows that their promoters contain at least two additional promoter elements. These lie on either side of the CHOP element and are conserved in all of these genes. Mutation of these elements substantially reduced the mtUPR responsiveness of the promoters suggesting that these elements, along with CHOP, coordinately regulate the mtUPR.

## Results

### Bioinformatic analysis of CHOP elements

We previously identified the CHOP consensus sequence as an mtUPR element in the *cpn60/10* promoter region [12]. Moreover, the other mtUPR responsive genes (*clpp*and *mtdnaj*) also have a CHOP consensus sequence in their promoter regions. These results suggest that a CHOP consensus sequence is a key factor for regulation of genes encoding proteins involved in mitochondrial quality control. However, a large region, encompassing 75 bp around the CHOP site contains two additional highly conserved regions. As described in the Supplementary Material (Supplemental Methods S1), we established a data base of 26599 genes and promoters from the UCSC Genome Bioinformatics site. We then searched this list to determine how many independent genes have a CHOP consensus in their promoter regions (see Supplemental Data S1). As shown in Table 1, a CHOP element was found in 3522 of the independent genes. We also searched a data base of 1042 genes encoding mitochondrial proteins in mammalian cells [14](Supplemental Data S3) and found 147 genes with a CHOP element and 131 genes from a data base of 1326 independent genes not coding for mitochondrial proteins [14](Supplemental Data S3).

**Table 1:** Number of Human genes with CHOP, MURE1 and 2 elements

Motifs	Set1: 26599 Genes	Set2: 1042 Mito Genes	Set3: 1326 Non-Mito Genes
CHOP(1)	3522 genes–13.8%	147 genes–14.1%	131 genes–9.9%
MURE1(2)	1340 genes–5%	63 genes–6%	57 genes–4.3%
MURE2(3)	3308 genes–12.4%	156 genes–15.0%	140 genes–10.6%
MURE1–CHOP–MURE2	481 genes–1.8%	23 genes–2.2%	21 genes–1.6%

(1) CHOP sequence: GG/ATTGCA

(2) MURE1 sequence: AGAATC/G/TGCT

(3) MURE2 sequence: GC/TACC/G/TCC/GAG

In searching for the motif group MURE1-CHOP-MURE2, outliers (>2 bp from average offset) were removed.

• Set1 consists of a large set of 1000 bp promoter regions from 26599 genes spanning most of the human genome. (Supplemental Data S2)

• Set2 consists of a set of 1000 bp promoter regions from a set of 1042 predicted mitochondrial genes [14]

• Set3 consists of a set of 1000 bp promoter regions from a set of 1326 non-mitochondrial genes taken from [14]

### Survey of the promoters of genes involved in mitochondrial protein control

**Figure 1:**
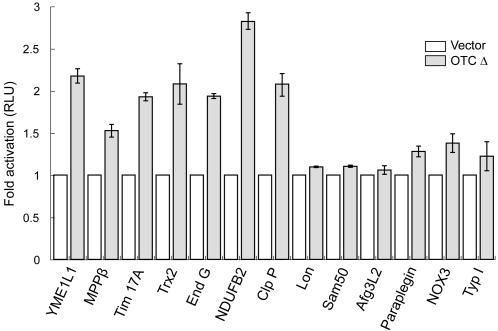
Survey of promoters responsive to mtUPR. Reporter constructs for mitochondrial proteins YME1L1, MPPβ (mitochondrial processing peptidase β subunit), Trx2 (mitochondrial thioredoxin), Tim17A, End G (Endonuclease G), NDUFB2 (subunit of complex I), and ClpP, which have CHOP consensus, and Lon, Sam50, Afg3L2, Paraplegin without CHOP consensus, or NOX3 (NADPH oxidases 3) and Typ I (Type I iodothyronine deiodinase), which are non-mitochondrial proteins but have CHOP consensus sequence in the promoter region, were tested. COS-7 cells were co-transfected with vector or vector containing OTCΔ and promoter-luciferase constructs and were used for luciferase assay 32 h after transfection. Data represent the mean±SEM from experiments performed in triplicate.

Since an improbably large number of both mitochondrial genes and non-mitochondrial genes contain the CHOP element, we determined whether the presence of such an element was sufficient to make the gene mtUPR responsive. As shown in Figure 1, we tested 13 genes, using promoter-luciferase constructs in transfected COS-7 cells, seven of which encode mitochondrial proteins and contain the CHOP element in their promoter and four for mitochondrial proteins but which do not contain this element. All seven of the genes with the CHOP element (genes for proteins YME1L1, ClpP and MPPβ, protein import component TIM17A, Complex I subunit NDUFB2, Endonuclease G and Thioredoxin 2) were mtUPR responsive, whereas all four lacking the element (genes for proteins AFG3L2, Paraplegin, Lon, and protein import factor Sam50) were not responsive. However, the presence of a CHOP element was not sufficient for mtUPR responsiveness as genes for non-mitochondrial proteins (Nox3 (NADPH oxidase 3) and Typ I, (Type I iodothyronine deiodinase)), which contain the CHOP element in their promoter regions were not up-regulated. As summarized in Table 2, we have so far discovered 11 genes which are mtUPR responsive and all contain the CHOP element in their promoters.

**Table 2:** Proteins regulated by the mtUPR

	Chaperone	Protease	Other
**Upregulated**	Cpn60/10 *	ClpP*	Tim17A
	MtDnaJ	YME1L1	NDUFB2
	MPPβ		CARD12
	Trx 2		Endonuclease G
			Cytochrome C reductase
**Not Upregulated**	mtHsp70 *	Paraplegin	Sam50
	Hsp72 *	Afg3L2	Tom20
	Bip *	Lon	Nox3
	Grp94 *		Typ I
	Calreticulin *		
	Calnexin *		
	PDI *		

* Previous data: Zhao et al. 2002 [12]

### Discovery of additional conserved elements neighboring the CHOP element

**Figure 2:**
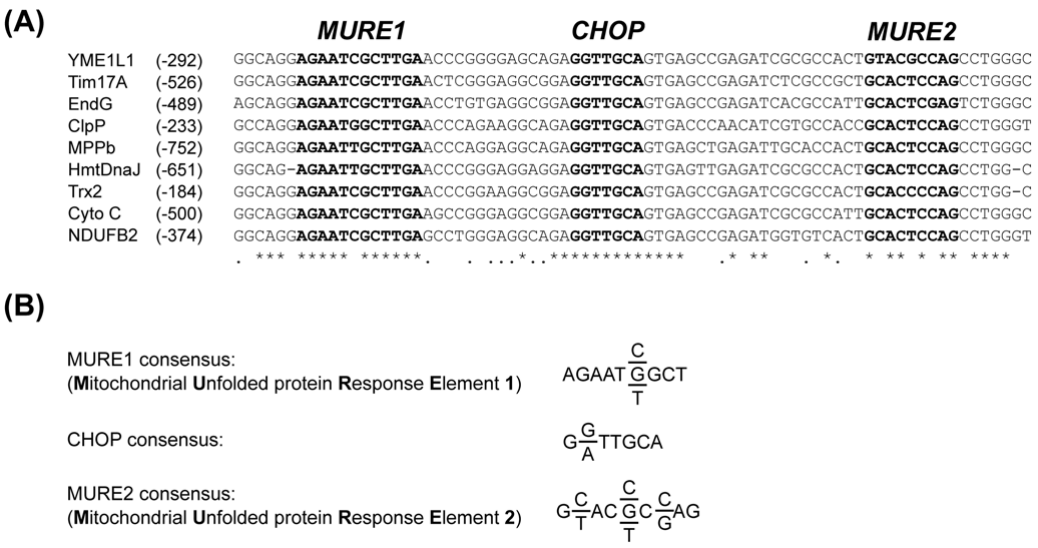
Nucleotide sequence alignment of the promoter region of mtUPR responsive genes. (A): mtUPR Genes, indicating the position in the promoter relative to the transcription start site, are shown on the left hand side of the figure. Asterisks identify identical sequences and bold letters show the highly conserved elements MURE1, CHOP, and MURE2. (B): Consensus sequence of the MURE1 and MURE2 elements, as well as the sequence for CHOP taken from [12].

When we compared the promoters of genes experimentally identified as being mtUPR responsive, we discovered a conserved region of approximately 100–150 bp around the CHOP consensus site (Fig. 2A). A region of 76 bp around CHOP/CEBPβ element was especially highly conserved in all but the Cpn60/10 gene. We identified an additional two putative elements in addition to the CHOP element (Fig. 2B) and named the first element MURE1 (**M**itochondrial **U**nfolded Protein **R**esponse **E**lement 1). MURE1 is a 12 bp element that is 14 bp upstream of the CHOP site. The second element, MURE2 consists of 9 bp and is 21 bp downstream of the CHOP element (Fig. 2A). These observations suggest that this highly conserved region containing the MURE1, CHOP and MURE2 sites may be significant for the regulation of genes involved in mitochondrial protein quality control.

### Effect of mutation of the MURE1 and MURE2 sites

**Figure 3:**
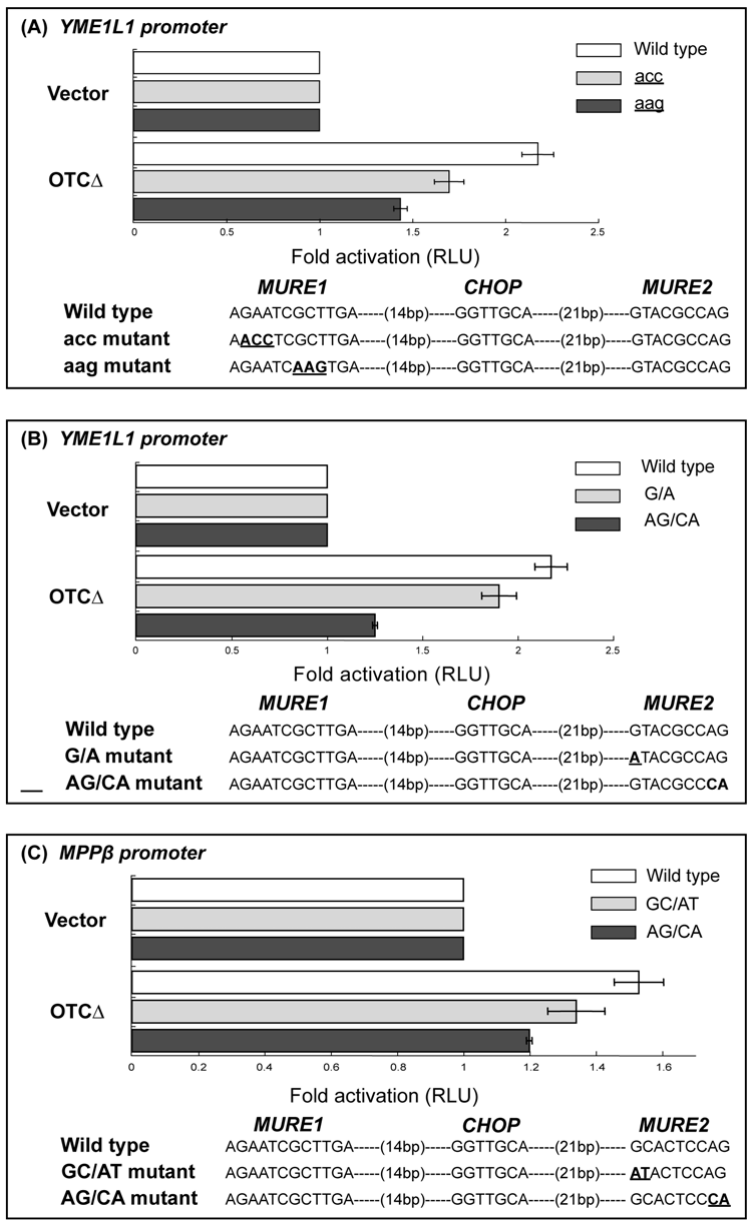
Effect of the mutation on MURE1 and MURE2 elements on mtUPR responsiveness. A. Effect of MURE1 mutations on *yme1l1* promoter activity.; B. Effect of MURE2 mutations on *yme1l1* promoter activity; C. Effect of MURE2 mutations on *mppβ* promoter activity. Luciferase activity is compared for each mutation in cells co-transfected with empty vector or vector- OTCΔ and the promoter reporter constructs and is expressed as the activity relative to that obtained for the wild type promoter (RLU-relative luciferase units). The results represent the mean±SEM for 3 independent experiments. The promoter sequence for wild type and mutants is shown.

To experimentally test the prediction that the MURE1 and MURE2 sites are involved in mtUPR, we created mutations in these sites in the *yme1l1* promoter and tested the effect on the inducibility of a promoter-luciferase construct in response to mtUPR. As shown in Figure 3A, both of the mutations: ***acc*** (A**GAA**TCGCTTGA to A**ACC**TCGCTTGA) and ***aag*** (AGAATC**GCT**TGA to AGAATC**AAG**TGA) in MURE1 affected the activation of the *yme1l1* promoter by mtUPR, with the ***aag*** mutation having a somewhat greater effect than the ***acc*** mutation (Fig. 3A). In the mutational analysis of the MURE2 site, we focused on the highly conserved nucleotides, namely the G in the first position and the AG in the last position in the *yme1l1* promoter (Fig. 3B), and created the mutants, ***g/a*** (**G**TACGCCAG to **A**TACGCCAG) and ***ag/ca*** mutant (GTACGCC**AG** to GTACGCC**CA**). In the *mppβ* promoter we created the mutations ***gc/at*** (**GC**ACTCCAG to **AT**ACTCCAG) and ***ag/ca*** (**GC**ACTCCAG to GCACTCC**CA**) (Fig.3C). We then tested the effects of these mutations on the activation of the *yme1l1*and *mppβ* promoter-luciferase constructs by mtUPR. The mutations ***g/a*** (*yme1l1*) and ***gc/at*** (*mppβ*) at the beginning of the MURE2 site had only a minor effect on both promoters, whereas the mutation ***ag/ca*** substantially affected the activation of these promoters by mtUPR (Figure 3B and C). Since we have previously shown the CHOP site to be required for mtUPR responsiveness [12], these observations indicate that at least these three elements are required for the activation and regulation of the mtUPR.

### Spacing of MURE1, CHOP and MURE2 elements

**Figure 4:**
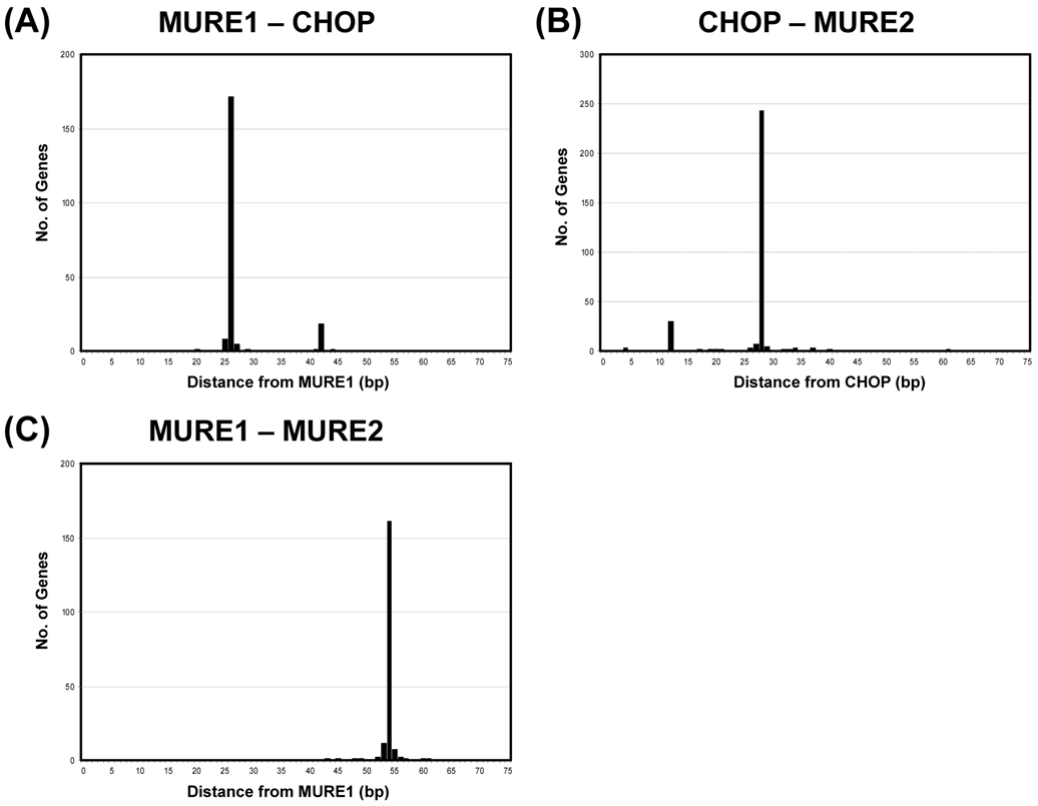
Spacing of MURE1, CHOP and MURE2 motifs. MURE1-CHOP (A), CHOP-MURE2 (B) and MURE1-MURE2 (C). Each figure shows the number of motif pairs with the given separation found in the set of predicted mitochondrial proteins. Separation is defined as the distance from the first base of the first motif to the first base of the second motif.

With the evidence that the three promoter elements MURE1, CHOP and MURE2 are required for mtUPR, we examined the frequency of this triplet of elements in the set of all independent genes, the mitochondrial genes and the non-mitochondrial genes. From this we found that although each individual element was very common (Table 1), the triplet was found in only 481 of the independent genes (1.8%) and only 23 of the mitochondrial genes (2.2%), and 21 of the non-mitochondrial genes (1.6%) (Table 1). Not only is the combination of these three elements highly correlated with mtUPR, but the spacing between these elements also appears to be highly conserved in the group of 9 genes shown experimentally to be responsive to mtUPR (Fig 2A). We therefore determine the spacing between the three elements, relative to each other, for all the genes containing the 3 elements in the mitochondrial database. In this analysis, shown in Figure 4, the spacing is defined as the distance in base pairs between the first nucleotide in each element (as defined in Fig. 2B). As shown there was very little variation in the spacing between the MURE1 element and the CHOP element, with the predominant spacing being 28 bp. The two outer elements, MURE1 and MURE2 also had very tight spacing at 54 bp. These results suggest that the separation between all three elements is significant for the interaction or discrimination of the transcription factors which bind to these sites. So far we have yet to identify the transcription factors which bind the MURE1 and MURE2 elements.

**Figure 5:**
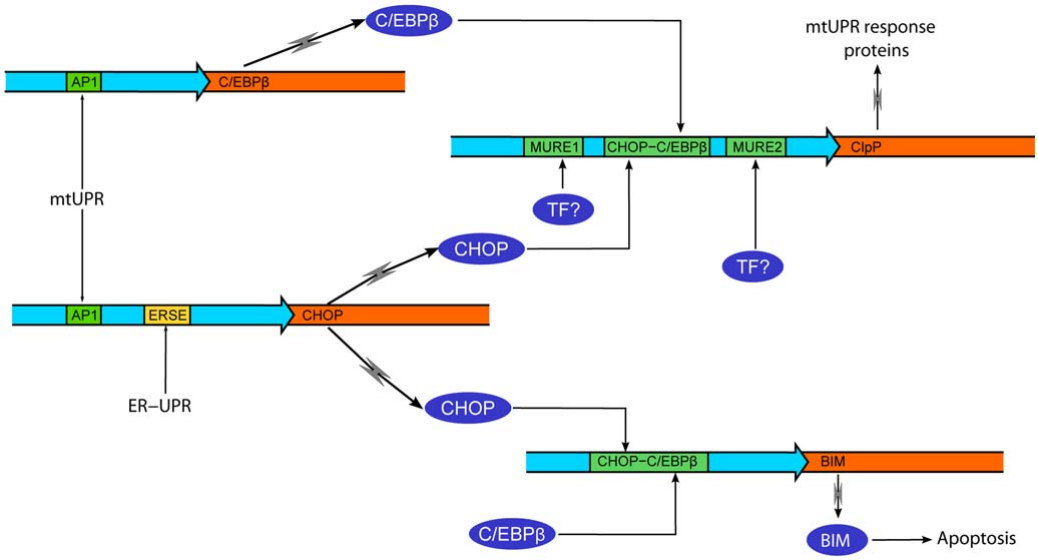
Comparison of regulatory circuits for mtUPR and erUPR. MtUPR activates transcription of both *chop* and *c/ebpβ* genes through an AP-1 site. These 2 transcription factors then bind to a CHOP element in the mtUPR responsive genes, along with transcription factors of unknown identity which bind to the MURE1 and 2 elements. ErUPR also activates transcription of CHOP (but not C/EBP genes) through an ER response element (ERSE). The function of CHOP in erUPR appears to be restricted to the induction of apoptosis through transcriptional activation of BIM.

## Discussion

We previously identified that the CHOP element (GG/ATTGCA) is involved in mtUPR and also that both erUPR and mtUPR induce the transcription of CHOP [12]. The induction of CHOP in erUPR has been shown to be through a cis-acting element in the CHOP promoter [8]. However, the induction of erUPR, by tunicamycin or thapsigargin does not significantly affect the induction of the mtUPR inducible gene for Cpn60, which has a CHOP element in its promoter [12]. In the accompanying paper [13], we reported on the identification of an AP1 promoter element in the CHOP gene through which transcription is regulated in response to mtUPR. We also report on the presence of an AP1 site in C/EBPβ, but not in C/EBPα. However, the specificity of induction of either erUPR or mtUPR genes cannot be explained purely on the basis of the induction of CHOP (and CEBPβ), and suggests that other factors are also required for the regulation of genes encoding mitochondrial proteins involved in mtUPR. In this report, we investigated the promoter region of genes which are up-regulated by mtUPR, and show that a CHOP element appears to be shared by all mtUPR responsive genes. However, the widespread presence of this element in the human genome suggests that this element alone cannot account for mtUPR, a fact supported by experiments with a range of promoter-reporter constructs (Table 2). A feature of mtUPR responsive genes appears to be that they contain a rather large conserved promoter region around the CHOP site. Using a collection of three databases, one representing the entire predicted open reading frames of the human genome (see Supplemental Data S2), a second consisting of a set of human genes encoding predicted mitochondrial proteins [14](Supplemental Data S3) and third, a set of genes encoding proteins predicted not to be mitochondrial [14](Supplemental Data S3), we searched for the frequency distribution of promoter elements predicted to occur in the conserved sequence around CHOP and found that at least two additional elements, MURE1 and MURE2 contribute to the regulation of all but one (Cpn60/10) of these genes.

Since both erUPR and mtUPR leads to increased levels of CHOP, the specificity of the mtUPR cannot be explained by the binding of CHOP and C/EBPβ alone. Specificity of mtUPR could be determined by activation of the MURE1 and/or MURE2 transcription factors in response to the stress dependent signaling from mitochondria to the nucleus (Fig. 5). Similarly, specificity of erUPR resides in the activation of ATF6 and NFY transcription factors [7], [15]. Conversely, since CHOP plus C/EBPα or β activates transcription of the *bim* gene [9], it is very likely that mtUPR, like erUPR, will lead to apoptosis.

So far the identity of the transcription factors which bind to these elements is only known for the CHOP element (CHOP and C/EBPβ). This element was originally discovered through its involvement in the response to UV damage [8]. The MURE1 element shows some similarity to a newly predicted motif in human promoter region, found using a homology search between human, mouse, rat and dog [16]. This sequence, C/T TAA C/T NGCT (N168), was predicted to be a novel transcriptional element but no transcription factor has yet been identified which binds to this element. MURE2 has similarity to the TTF1 (Thyroid Transcription Factor 1) binding site (GCNCTNNAG) [17]. The TTF1 transcription factor has been characterized. It is a homeodomain-containing nuclear transcription factor and is expressed in the epithelium of the developing lung, thyroid, and in the central nervous system [18]. TTF-1 is critical for normal lung formation, since *T/ebp* null mutant mice lacking TTF1 die at birth from a lack of peripheral lung tissues [19]. AS explained above, it is possible that the specificity of mtUPR resides in the MURE1 and MURE2 transcription factors. They may be activated by covalent modification in response to the accumulation of unfolded proteins in the mitochondrial matrix or this may lead to transcriptional activation of genes encoding MURE1 and 2, a process that would require the intervention of yet another transcription factor. Since a different regulatory circuit has been suggested for mtUPR in *c.elegans*, it is possible that this regulatory pathway may also exist in mammalian cells and be integrated into the CHOP/MURE1 and 2 pathways.

Interestingly, the number of combinations of all three of these motifs is present only in 1.8% of all human genes (Table 1 and see Supplemental Data S1 for the identity of these genes). However, this still represents 481 independent genes. This is not dissimilar to the finding in erUPR where a similarly large suite of genes is transcriptionally activated by the accumulation of unfolded proteins in the ER lumen [20]–[22]. These genes encode proteins that are involved in protein quality control as well as proteins required for the maintenance of the secretory pathway. The functional role of most of the 481 genes which contain the 3 elements is unknown; many of them encode non-mitochondrial proteins or proteins of unknown function. The list of 23 mitochondrial proteins with the triple element is also given in Supplemental Data S1. Besides proteins involved in mitochondrial protein control, they also include subunits of the oxidative phosphorylation machinery, and a number of enzymes involved in oxidative metabolism. The supplementary information also provides the identity of the genes and promoter elements, including the gene position and the offset from the transcription start site of the 3 elements (Supplemental Data S1)

Although the role of many of the predicted gene products elevated by mtUPR is unclear, one interesting observation is the activation of *yme1l1*, which encodes an AAA superfamily metalloprotease and has both chaperone and protease activity [23]. There are three ATP regulated proteins (YME1L1, Afg3L2, and Paraplegin) which are located in inner membrane of mitochondria [23]. Interestingly, the active site of YME1L1 is located in the inter-membrane space in contrast to Afg3L2 and Paraplegin whose active sites are in the matrix, yet only YME1L1 is activated by mtUPR (Table 2). However, this enzyme is unlikely to play a role in removing unfolded proteins from the matrix. Perhaps this protease of the inter-membrane space plays a role in removing unfolded proteins anchored in the inner membrane or in the modification of the inner membrane. The induction of Endonuclease G is also of interest as it is a nuclear encoded endonuclease, localized in the mitochondrial inter-membrane space [24] and its up-regulation may be important for caspase-independent apoptosis [25]. Upon induction of apoptosis Endonuclease G has been shown to be translocated to the nucleus at a time which coincides with large-scale DNA fragmentation [25], [26]. It cannot be excluded that a fraction of OTCΔ or some other unfolded inter-membrane space proteins accumulate in the inter-membrane space and that this leads to the release of Endonuclease G from the mitochondrion and mediates apoptotic cell death that is triggered by mtUPR.

Further characterization of mtUPR responsive gene products should provide insights on how cells discriminate organelle specific stress responses and balance survival with death under the condition of stress.

## Materials and Methods

### Materials

Tunicamycin was purchased from Sigma Chemical (St Louis, USA). Human genomic DNA was purchased from Promega (Madison, USA). All reagents were of reagent grade quality.

### Plasmid construction, transfection and promoter analysis

MtUPR was created with expression vector containing DNA encoding a deletion mutant (OTCΔ) of the mitochondrial matrix enzyme ornithine transcarbamylase (OTC) and was constructed as described previously [12]. Promoters used in reporter constructs are based on the human genome sequence information of NCBI. Promoter regions were amplified by PCR using human genomic DNA as template and consisted of 1000 bp region upstream of the transcription start-site. Promoter DNA was cloned into the pGL3-Basic vector (Promega, Madison, USA.), alongside a promoterless firefly luciferase coding sequence. Cos-7 cells were cultured in DME/5 % fetal calf serum and transfected at 90 % confluence using Lipofectamine 2000 (Invitrogen). Promoter analysis using luciferase assay was carried out as described previously [12]. To induce erUPR, cells were treated with 2 µg/ml tunicamycin for 10 h.

### Promoter element pair searches

A data base of 26599 independent genes and promoters was downloaded from the UCSC Genome Bioinformatics site, and divided into mitochondrial, non-mitochondrial and general sets based on the protein data bases described in [14]. These sets were searched for known or predicted promoter elements, and those elements that occurred regularly in close proximity were extracted and details of the average separation, the standard deviation of the separation and the number of pairs found were recorded. This data was searched for those motif pairs related to the CHOP promoter element. Detailed descriptions of the construction of the data bases and the searching algorithms used are given in the Supplementary Documentation.

## Supporting Information

Methods S1 Overview of supplied supplemental data and detailed algorithm used to identify related/neighbouring promoter elements.
(0.42 MB DOC)

Data S1 A list of mitochondrial, non-mitochonrial and general genes which contain mtUPR promoter elements.
(0.12 MB XLS)

Data S2 A list of "independent" genes and related original gene records used in the search algorithm
(5.81 MB XLS)

Data S3 The list of predicted mitochondrial and non-mitochondrial "independent" genes used in the search algorithm
(0.58 MB XLS)
