# Supplementary material for: Discovery of Genes Activated by the Mitochondrial Unfolded Protein Response (mtUPR) and Cognate Promoter Elements
Source: PLoS One. 2007 Sep 12;2(9):e874. doi: 10.1371/journal.pone.0000874 (PMC1964532; doi:10.1371/journal.pone.0000874)
Supplement: Methods S1 — Overview of supplied supplemental data and detailed algorithm used to identify related/neighbouring promoter elements. (0.42 MB DOC) [file pone.0000874.s001.doc]

Supplemental Data for

“Discovery of Genes Activated by the Mitochondrial Unfolded Protein Response (mtUPR) and Cognate Promoter Elements.”

The supplemental data supplied consists of:

1. This document that lists the supplemental data and the algorithm used to identify related/neighbouring promoter elements,
2. A list of genes/proteins containing the promoter element triplet MURE1-CHOP-MURE2 in the first 1000 base pairs of the promoter region. Given in the Excel file *Supplemental Data S2.xls*
3. A list of “independent” genes and related original gene records used in the algorithm given in the Excel file: *Supplemental Data S3.xls*
4. The list of predicted mitochondrial and non-mitochondrial “independent” genes used in the algorithm given in the Excel file: *Supplemental Data S4.xls*

Any additional data including source files for any programs used, datasets for the promoters, discovered genes and discovered promoter element pairs can be supplied on request to [J.Aldridge@latrobe.edu.au](mailto:J.Aldridge@latrobe.edu.au) or [N.Hoogenraad@latrobe.edu.au](mailto:N.Hoogenraad@latrobe.edu.au) .

**Supplemental Methods S1: Associated motif discovery Algorithm:**

The algorithm used to identify related promoter elements is a two stage process. Stage one simply identifies all known or predicted promoter elements in a region around the transcription start site in a given set of genes. The second stage involves analysing the positions of these discovered motifs and searching for commonly appearing motif pairs with fixed separation, as would be expected for the binding of transcription protein complexes.

**Stage 1 details: Motif discovery.**

To perform the motif discovery search, two sets of data were required: First, a list of motifs to search for, and second, a list of genes to search in.

**Promoter element list**

The first requirement was to collect a comprehensive list of promoter element consensus sequences that could then be used to search given promoter/gene sequences. This list of motifs was collected as follows:

A set of 129 elements was obtained from the Transfac database [s1] by searching the Transfac “SITE” database table with the condition “description field = ‘consensus’”, then manually curating the results for homo-sapien associated motifs.

A further 43 motifs from the Jaspar Database [s2] were obtained by downloading the matrix representations of the promoter elements with “Species = ‘Homo sapiens’”.

These matrix representation were converted to consensus representations using the same algorithm used in the consensus calculation from the transfac site: (ref <http://www.gene-regulation.com/pub/databases/transfac/doc/matrix1.html>)

That is:

*...a single nucleotide is shown if its frequency is greater than 50% and at least twice as high as the second most frequent nucleotide. A double-degenerate code indicates that the corresponding two nucleotides occur in more than 75% of the underlying sequences but each of them is present in less than 50%. Usage of triple-degenerate codes is restricted to those positions where one of the nucleotides did not show up at all in the sequence set and none of the afore-mentioned rules applies. All other frequency distributions are represented by the letter "N".*

The final set of motifs used in the search came from the paper *“Systematic discovery of regulatory motifs in human promoters and 3’ UTRs by comparison of several mammals”* by Xiaohui Xie et al. [s3] where they used homology search between human, mouse, rat and dog gene sequences to identify potential promoter elements. The final concatenated list of elements is given in the Supplemental Appendix A.

**Promoter/Gene sequences**

The second data set required is a set of promoter regions to be searched. This was obtained from the UCSC Genome Bioinformatics site, [http://genome.ucsc.edu/](http://genome.ucsc.edu/index.html) [s4] where the gene and promoter sequence for all known and predicted human genes was downloaded , using the Table Browser interface [s5]. The sequence downloaded consisted of 5000bp upstream of the transcription start site to the end of the coding sequence. This set of genes contained a high number of duplicate and redundant entries whose transcripts and promoter regions overlapped, so we filtered the gene set to select a single representative gene/promoter region for those whose transcription start site was within approximately 50 base pairs.

The exact filtering algorithm was:

1. For a given chromosome, find the first gene and make this the start of a list.
2. Find the next gene, and if its transcription start site (TSS) is within 50bp of the average TSS for the list, add it to the end of the list and recalculate the average TSS position, then repeat this step.
3. Once a gene is found whose TSS is greater than 50bp from the list average, record the details of the existing list (not including the latest gene), then use the new gene as the start of a new list, return to step 2.
4. Stop when the end of the chromosome is reached.
5. For each list of related genes, pick and record a single representative gene.

This gave a list of 26,598 independent genes provided in the supplementary data file *“Supplemental Data S3”*. From each of these genes, a region ±2000bp around the transcription start site was selected to be searched with all the elements from the above consensus list.

Also required were subsets of this global list of independent genes; namely a set of mitochondrial and non-mitochondrial genes. These were obtained from those given in the paper *“Systematic identification of human mitochondrial disease genes through integrative genomics.”* [s6]. Starting with their list of mitochondrial and non-mitochondrial proteins, the associated independent gene from the global list was extracted and a collection of 1,042 mitochondrial associated independent genes and 1,326 non-mitochondrial independent genes was collected (See Supplemental Data S4 for the list of these independent genes and their associated original genes)

**Motif Discovery**

The next step was the actual searching of the gene sequences for promoter elements. To do this we wrote a java program to search through all the global promoter/gene regions for each of the listed promoter elements, getting a list of 8,296,530 potential motifs across the 26,598 genes.

Mitochondrial and Non-Mitochondrial subsets of this global motif list were then created by extracting those motifs found in the mitochondrial and non-mitochondrial genes.

**Stage 2 details: Motif Pair discovery.**

The next step in the process was to identify what promoter elements appear in regular close proximity to each other.

This was done by stepping through all the motifs discovered and recording the details of every pair of motifs found within 75bp of each other (measured from start of first motif to start of second motif).

The details recorded included:

1. The first motif description,
2. the direction(strand) for the first motif relative to the gene of interest,
3. the second motif description,
4. the direction(strand) for the second motif relative to the gene of interest,
5. the number of times the motif pair was found per 100 genes
6. a number representing how common the motif pairing is in relation to the individual motifs, calculated as follows:
   The average of: the percentage of the first motifs that were followed by the second motif and the percentage of the second motifs that were preceded with the first motif.
7. the average offset of the start of the second motif from the start of the first motif,
8. the standard deviation of the second motif offset from the first motif,
9. a score for the pair computed from some of the above values and given below,
10. the number of motif pairs found at each separation distance (from 0bp to 75bp)

The score for a motif pair: Motif1 – Motif2 (number 9 in the above list), is calculated from:

1. The standard deviation of the position offset between Motif1 and Motif2,
2. the total number of this motif pair found per 100 genes,
3. the ratio of motif pairs to the individual motifs that make up the pair.

The base of the calculation of the score consists of three computed variables: **A**, **B** and **C**:

**Variable A** reflects how common the motif pairing is in relation to the individual motifs and is taken directly from data value 6 in the above list.

**Variable B** reflects the fact that a transcription factor binding complex should touch the DNA strand at fixed points, therefore the separation of the binding motifs should be approximately constant. This is expressed by setting Variable B as one over the standard deviation of the motif offset (data value 8) plus 1; or:

**B = 1/(SD + 1)**

Where SD = data value 8 in the above list. (The “plus one” simply prevents a divide by zero.)

**Variable C** is defined as log base 10 of the number of motif pairs found per 100 genes; or:

**C = Log_10_(data value 5)**

This log_10_ value was chosen due to the fact that if we used the raw number of motif pairs found, then those motif pairs related to cell systems/organelles with lots of associated genes would massively outscore pairs related to more limited cell systems. Thus the log base 10 was chosen as a compromise between counting the number of motif pairs as significant without overwhelming pairs from limited sets of genes.

The final score is then simply the multiplication of these three variables.

**Score = A x B x C.**

Thus from this list of promoter element pairs, the motifs that appear in regular close proximity can easily be identified as those having the highest score.

**Additional Complications:**

One problem that came up in finding these pairs was the cases where the same pair could have two distinct, active offsets. For example, for a given motif pair, the graph of the offsets discovered could look like

0

20

40

60

80

100

120

1

4

7

10

13

16

19

22

25

28

31

34

37

40

43

46

49

52

55

58

61

64

67

70

73

Thus in cases like this, simply finding the average and standard deviation of the offset completely hides the fact that this is actually two distinct binding site pairs.

Thus an algorithm was developed to distinguish motif distributions that represented multiple distinct binding patterns from those with only a single motif offset position. This general algorithm is as follows:

1. A minimum height for the potential peaks is assigned. A value of six times the average height was found to be effective:

0

20

40

60

80

100

120

1

4

7

10

13

16

19

22

25

28

31

34

37

40

43

46

49

52

55

58

61

64

67

70

73

1. Find highest point above this, and record the data points within three bp of peak

0

20

40

60

80

100

120

1

4

7

10

13

16

19

22

25

28

31

34

37

40

43

46

49

52

55

58

61

64

67

70

73

1. Set height of this point and area around it (+-3bp) to zero in the motif offset array. (This cuts out this area for further searches)

0

20

40

60

80

100

120

1

4

7

10

13

16

19

22

25

28

31

34

37

40

43

46

49

52

55

58

61

64

67

70

73

1. Repeat Step 2-4 on modified data to find remaining peaks

For each peak, the average peak position is taken by averaging over the three bp region around the peak, and the standard deviation is taken across the whole region with the other peaks (and their neighbours) removed. Note, this is now the standard deviation around the peak, NOT the standard deviation of the mean.

All the other data as listed in the description of Stage 2 is recorded for each peak.

Referencess

**s1.** Matys, V., E. Fricke, et al. (2003). "TRANSFAC: transcriptional regulation, from patterns to profiles." Nucleic Acids Res **31**(1): 374-8.

s2. Sandelin, A., W. Alkema, et al. (2004). "JASPAR: an open-access database for eukaryotic transcription factor binding profiles." Nucleic Acids Res **32**(Database issue): D91-4.

s3. Xie, X., J. Lu, et al. (2005). "Systematic discovery of regulatory motifs in human promoters and 3' UTRs by comparison of several mammals." Nature **434**(7031): 338-45.

s4. Kent, W.J., Sugnet, C. W., Furey, T. S., Roskin, K.M., Pringle, T. H., Zahler, A. M., and Haussler, D. [The Human Genome Browser at UCSC](http://www.genome.org/cgi/content/abstract/12/6/996). Genome Res. **12**(6), 996-1006 (2002).

s5. Karolchik, D., Hinrichs, A.S., Furey, T.S., Roskin, K.M., Sugnet, C.W., Haussler, D. and Kent, W.J. [The UCSC Table Browser data retrieval tool](http://nar.oupjournals.org/cgi/content/abstract/32/suppl_1/D493?ijkey=06tIQcBr2VZNz&keytype=ref). Nucl. Acids Res. **32**(Suppl 1), D493-D496 (2004).

s6. Calvo, S., M. Jain, et al. (2006). "Systematic identification of human mitochondrial disease genes through integrative genomics." Nat Genet **38**(5): 576-82.

**Supplemental Appendix A: Promoter element consensus list**

| **Consensus** | **Name and source** |
| --- | --- |
| AAAGAAATTCC | CD28RC, Transfac |
| AAAYRnCTG | N77, Xie X, et al. |
| AAAYWAACM | HFH-4, Xie X, et al. |
| AAAnWWTGC | N144, Xie X, et al. |
| AACAAACACAAA | H1TF1, Transfac |
| AACAATRG | SOX-9, Jaspar |
| AACTTT | IRF1(*), Xie X, et al. |
| AACWWCAAnK | FAC1(*), Xie X, et al. |
| AACYnnnnTTCCS | N113, Xie X, et al. |
| AAGATAAAACC | RVF, Transfac |
| AAGTGAAAGT | PRDIBF1, Transfac |
| AAGTGTTTGC | TGT3, Transfac |
| AAGWWRnYGGC | N63, Xie X, et al. |
| AAGYATGCA | TEF1, Transfac |
| ACACCCAAATATGGGCGAC | CBF, Transfac |
| ACAWYAAAG | N142, Xie X, et al. |
| ACAWnRnSRCGG | N103, Xie X, et al. |
| ACCGGAAGNS | NRF-2, Jaspar |
| ACCGGAAGT | SAP-1, Jaspar |
| ACCTGTTG | N57, Xie X, et al. |
| ACTAYRnnnCCCR | N4, Xie X, et al. |
| ACTWSnACTnY | N105, Xie X, et al. |
| AGAACANNNTGTTCT | AR_01, GR_01, MR, PR, Transfac |
| AGAGGAACT | PU1, Transfac |
| AGCYRWTTC | N135, Xie X, et al. |
| AGGAAR | PEA3, Transfac |
| AGGTCANNNAGGTCA | VDR_02, Transfac |
| AGGTCANNNTGACCT | ER, Transfac |
| AGGTCATGACCT | RAR, TR_02, VDR_01, Transfac |
| AMCATCTGKT | Tal1beta-E47S, Jaspar |
| ANANTTTCC | NPTCII, Transfac |
| ANCACGTG | Max, Jaspar |
| ANNGTAAACAA | FREAC2_01, Transfac |
| ANSGTAAACAA | FREAC-2, Jaspar |
| ARGGGTTAA | FXR(*), Xie X, et al. |
| ATCAATCAAW | Pbx, Jaspar |
| ATCMnTCCGY | N99, Xie X, et al. |
| ATGACTCAGCANTTNNG | TCF11/MAFG_01, Transfac |
| ATGCAAAT | OCT1_01, OCT2, OCT6, Transfac |
| ATGCWAAT | OCT4, Transfac |
| ATGGYGGA | N102, Xie X, et al. |
| ATTC[1:30]GCCA | HIP1, Transfac |
| ATTTNNNNATTT | HINFA, Transfac |
| ATTTTTCTGATTGGCCAAAG | YB1, Transfac |
| AWNNAGGTCA | RORalfa-1, Jaspar |
| BNKGNTGACGY | CREB, Jaspar |
| CAACTGAC | MYOD_01, Transfac |
| CACGTG | MYC, Transfac, Xie X, et al. |
| CACGTGR | USF, Jaspar |
| CAGCCCCCGCGCAGC | ETF, Transfac |
| CAGCTG | AP-4, Xie X, et al. |
| CAGGTA | AREB6, Xie X, et al. |
| CAGGTG | E12, Xie X, et al. |
| CAGnWMCnnnGAC | N143, Xie X, et al. |
| CAGnYGKnAAA | N160, Xie X, et al. |
| CATRRAGC | N134, Xie X, et al. |
| CATTGTYY | SOX-9, Xie X, et al. |
| CATYAS | IUF1_01, Transfac |
| CCAATnnSnnnGCG | N104, Xie X, et al. |
| CCAWWnAAGG | SRF, Xie X, et al. |
| CCAWYnnGAAR | N124, Xie X, et al. |
| CCAWnWWnnnGGC | N165, Xie X, et al. |
| CCAnnAGRKGGC | N45, Xie X, et al. |
| CCCCMAAMCAMCCMCMMMC | RREB-1, Jaspar |
| CCCCTAGCAACAGATG | RFX, Transfac |
| CCCMNSSS | AP2_01, Transfac |
| CCCnnGGGAR | OLF-1, Xie X, et al. |
| CCCnnnnnnAAGWT | N158, Xie X, et al. |
| CCGCCCCCGC | EGR2, Transfac |
| CCGGAAR | Elk-1, Jaspar |
| CCGnMnnTnACG | N106, Xie X, et al. |
| CCRAAGACCACCCACAATGATGGT | GLI, Transfac |
| CCTnTMAGA | N167, Xie X, et al. |
| CCWTNTTNNNW | YY1_02, Transfac |
| CGCAGCTGCG | Hen-1, Jaspar |
| CGCCCCCGC | WTZFP, Transfac |
| CGCCCSCGC | EGR1, Transfac |
| CGGAAGTG | E4TF1, EF1A, Transfac |
| CGGAARnGGCnG | N81, Xie X, et al. |
| CGTSACG | PAX-3, Xie X, et al. |
| CNWNWCACGCWW | AHR_03, Transfac |
| CRGAARnnnnCGA | N100, Xie X, et al. |
| CTAATG | ISL1, Transfac |
| CTATTTWTAG | MEF2, Jaspar |
| CTAWWWATA | RSRFC4, Xie X, et al. |
| CTCATGA | OCT1_01, OCT2, Transfac |
| CTCTCTGG | UBP1, Transfac |
| CTCnAnGTGnY | N171, Xie X, et al. |
| CTGCAGY | N101, Xie X, et al. |
| CTGRYYYnATT | N153, Xie X, et al. |
| CTGYnnCTYTAA | N82, Xie X, et al. |
| CTTTAAR | N29, Xie X, et al. |
| CTTTATCTGG | ANF_01, Transfac |
| CTTTCAGTTT | ISGF1, Transfac |
| CTTTCTCTTT | ISGF2, Transfac |
| CTTTGW | LEF1, Xie X, et al. |
| CTWAWGTAAACANWG | FREAC4_01, Transfac |
| CYTAGCAAY | N34, Xie X, et al. |
| GAANNGAANNGAA | HSF, Transfac |
| GAAnYnYGACnY | N137, Xie X, et al. |
| GACGCANYGRWNNNMG | BSAP_01, Transfac |
| GATAAGR | GATA-X, Xie X, et al. |
| GATGKMRGCG | N148, Xie X, et al. |
| GATTGGY | NF-Y, Xie X, et al. |
| GCACCAATCACAGCGCGC | H1TF2, Transfac |
| GCAnCTGnY | MYOD, Xie X, et al. |
| GCCACATGACC | NFME3, TFE3, Transfac |
| GCCATnTTG | YY1, Xie X, et al. |
| GCCCATATAWGG | SRF, Jaspar |
| GCCNNNRGS | AP2alpha, Jaspar |
| GCCTGCAGGC | KER1, Transfac |
| GCCnnnWTAAR | N95, Xie X, et al. |
| GCGCCCTTTGGACCT | LIT1, Transfac |
| GCGGGGC | GCF_02, Transfac |
| GCGSCMnTTT | N164, Xie X, et al. |
| GCGTGGGCGKDK | WT1_01, Transfac |
| GCGnnAnTTCC | C-REL(*), Xie X, et al. |
| GCNCTNNAG | TTF1_02, Transfac |
| GCTTCAGTTT | ISGF3_01, Transfac |
| GCTnWTTGK | N78, Xie X, et al. |
| GGAACCTCCCCC | NTF, Transfac |
| GGAAnCGGAAnY | N21, Xie X, et al. |
| GGAGGAAAAACTGTTTCAT | NFAT, Transfac |
| GGAMTnnnnnTCCY | N74, Xie X, et al. |
| GGARnTKYCCA | N163, Xie X, et al. |
| GGATGTCCATATTAGGACATCT | SRF_01, Transfac |
| GGATTA | PITX2, Xie X, et al. |
| GGCCACGTGACC | TFEB, Transfac |
| GGCDGGGT | SP1, Jaspar |
| GGCKCATGS | N159, Xie X, et al. |
| GGCnKCCATnK | N88, Xie X, et al. |
| GGCnRnWCTTYS | N118, Xie X, et al. |
| GGCnnMSMYnTTG | N123, Xie X, et al. |
| GGGACTTTCC | EBP1, Transfac |
| GGGAGGRR | MAZ, Xie X, et al. |
| GGGAGTG | P300_01, Transfac |
| GGGAMTNYCC | NFKB_01, Transfac |
| GGGCGGNNNNGGGCGG | LSF*, Transfac |
| GGGCGGR | SP1, Xie X, et al. |
| GGGGGAGGG | H4TF1, Transfac |
| GGGGRTTCCCC | p50, Jaspar |
| GGGRATTTCC | p65, Jaspar |
| GGGTCAWNGRGTTCA | RXR-VDR, Jaspar |
| GGGTGGG | PUF, Transfac |
| GGGTGGRR | PAX-4, Xie X, et al. |
| GGGTGTGG | TEF2, Transfac |
| GGGYGTGnY | N31, Xie X, et al. |
| GGGnRMnnYCAT | N119, Xie X, et al. |
| GGGnnTTTCC | NF-KAPPAB, Xie X, et al. |
| GGNNGAGGGAGARRRR | PUR_01, Transfac |
| GGSCACRTGAC | USF, Transfac |
| GGTTCTCNNNNCGGTCCG | H4TF2, Transfac |
| GGTTCTTTCCGCC | LSF, Transfac |
| GKCGCnnnnnnnTGAYG | N19, Xie X, et al. |
| GNNCACTCAAG | TTF1_01, Transfac |
| GRGRTTKCAY | NFGMA, Transfac |
| GTAAACA | FREAC-4, Jaspar |
| GTAAATAAAGA | FREAC3_01, Transfac |
| GTCATNNW | TCF11_01, Transfac |
| GTCnYYATGR | N98, Xie X, et al. |
| GTGACGY | E4F1, Xie X, et al. |
| GTGGGTGK | N91, Xie X, et al. |
| GTGGWWWG | CEBP_01, Transfac |
| GTGTCAGTCA | NFE, Transfac |
| GTTAATNATTAAC | HNF1_01, VHNF1, Transfac |
| GTTGCYNGRCAAC | EFC, Transfac |
| GTTGnYnnRGnAAC | N43, Xie X, et al. |
| GTTRYCATRR | N37, Xie X, et al. |
| GTTnYYnnGGTnA | N89, Xie X, et al. |
| KAGGGGNA | MZF_5-13, Jaspar |
| KCCGnSWTTT | N157, Xie X, et al. |
| KGCWARGKYCAY | HNF4, Transfac |
| KMCATnnWGGA | N116, Xie X, et al. |
| KNNTRTTTRTTT | HFH-3, Jaspar |
| KNNTRTTTRTTTA | HF3_01, Transfac |
| KRCTCnnnnMAnAGC | N120, Xie X, et al. |
| KRGGCKRRK | SP1_01, Transfac |
| KTGGYRSGAA | N112, Xie X, et al. |
| MATNNNWAAT | BRN2_01, Transfac |
| MATNNWAAT | BRN2_01, Transfac |
| MATWAAT | BRN2_01, Transfac |
| MCAATnnnnnGCG | N110, Xie X, et al. |
| MGGAAGTG | GABP, Xie X, et al. |
| MRGCARCWGSWG | Myf, Jaspar |
| MSGGACATGYCCGGGCATGT | p53, Jaspar |
| MYAATnnnnnnnGGC | N138, Xie X, et al. |
| RAAAGYGAAACC | Irf-1, Jaspar |
| RAAGnYnnCTTY | N125, Xie X, et al. |
| RACCACAR | AML, Xie X, et al. |
| RACTnnRTTTnC | N66, Xie X, et al. |
| RASCACGTGGT | Myc-Max, Jaspar |
| RCAGNTG | E2A, Transfac |
| RCGCAnGCGY | NRF-1, Xie X, et al. |
| RGAAnnTTC | HSF1, Xie X, et al. |
| RGAGGAARY | PU.1, Xie X, et al. |
| RGTTAMWnATT | HNF-1, Xie X, et al. |
| RRAGTTGT | N129, Xie X, et al. |
| RRCCAATSRG | NFY_01, Transfac |
| RRCCGTTA | N146, Xie X, et al. |
| RTAAACA | FREAC-2, Xie X, et al. |
| RTCTGGMWT | Thing1-E47, Jaspar |
| RTKAYGTAAY | E4BP4_01, Transfac |
| RTTACRYAAT | HLF, Jaspar |
| RTTTnnnYTGGM | N107, Xie X, et al. |
| RYAAAKnnnnnnTTGW | N151, Xie X, et al. |
| RYCACnnRnnRnCAG | N128, Xie X, et al. |
| RYTAAWnnnTGAY | N133, Xie X, et al. |
| RYTGCnWTGGnR | N87, Xie X, et al. |
| RYTGCnnRGnAAC | MIF-1, Xie X, et al. |
| RYTTCCTG | C-ETS-2, Xie X, et al. |
| RnCTGnYnRnCTGnY | N154, Xie X, et al. |
| RnGTGGGC | N140, Xie X, et al. |
| RnTCAnnRnnYnATTW | N122, Xie X, et al. |
| SAGGAAGY | TCF2ALPHA, Transfac |
| SCGGAAGY | ELK-1, Xie X, et al. |
| SCGSSSC | GCF_01, Transfac |
| SGAAAGYGAAASCNWW | Irf-2, Jaspar |
| SGCGSSAAA | E2F-1/DP-2, Xie X, et al. |
| SGGRNWTTCC | c-REL, Jaspar |
| SMGGAWGY | ETS1, Transfac |
| SMTTTTGT | N62, Xie X, et al. |
| SSGCCATCTTSNCTS | YY1_03, Transfac |
| STAGGTCACNGTGACCYANT | PPARgamma, Jaspar |
| STTTCRnTTT | IRF, Xie X, et al. |
| SYATTGTG | N71, Xie X, et al. |
| SnACAnnnYSYAGA | N80, Xie X, et al. |
| TAATGARAT | OCT6, Transfac |
| TAATTA | CHX10, Xie X, et al. |
| TAAWWATAG | RSRFC4*, Xie X, et al. |
| TAAYnRnnTCC | N136, Xie X, et al. |
| TAAnnYSGCG | N162, Xie X, et al. |
| TATAAA | TATA, Xie X, et al., TBP, Transfac |
| TCAGGTCACAGTGACCTGA | H2RIIBP, Transfac |
| TCAGRTA | NFGMB, Transfac |
| TCAnnTGAY | SREBP-1, Xie X, et al. |
| TCCATTKW | N132, Xie X, et al. |
| TCCCCMNSSS | AP2_02, Transfac |
| TCCCRnnRTGC | N39, Xie X, et al. |
| TCNTACTC | CEBP_02, Transfac |
| TCTCTTA | MYC, Transfac |
| TGACAGnY | MEIS1, Xie X, et al. |
| TGACATY | N42, Xie X, et al. |
| TGACCTTG | ERRALPHA, Xie X, et al. |
| TGACCTY | ESRRA, Xie X, et al. |
| TGACGTAAC | E4F, Transfac |
| TGACGTMW | CREB_01, Transfac |
| TGACGTYA | CRPJUN_01, Transfac |
| TGACGYMR | ATF_01, Transfac |
| TGACTCAG | NFE2_01, Transfac |
| TGAMCTTTGMMCYT | COUP-TF, Jaspar |
| TGANCCCTTGACCCCT | ARP1_01, Transfac |
| TGASTMA | AP1, Transfac |
| TGASTMAGC | NF-E2, Xie X, et al. |
| TGATTTRY | GFI-1, Xie X, et al. |
| TGAYRTCA | ATF3, Xie X, et al. |
| TGAnTCA | AP-1, Xie X, et al. |
| TGAnnYRGCA | TCF11/MAFG, Xie X, et al. |
| TGCCAAR | NF-1, Xie X, et al. |
| TGCGCAnK | N22, Xie X, et al. |
| TGCGTGAGAAGA | AHR_01, Transfac |
| TGCTGAY | N92, Xie X, et al. |
| TGGAAA | NF-AT, Xie X, et al. |
| TGGAATGT | TEF1*, Transfac |
| TGGACYYNNNNTGGCCC | LFA1, Transfac |
| TGGACYYNNNTGGCCC | LFA1, Transfac |
| TGGACYYNNTGGCCC | LFA1, Transfac |
| TGGACYYNTGGCCC | LFA1, Transfac |
| TGGACYYTGGCCC | LFA1, Transfac |
| TGGGGATTCCCCA | H2TF1, HIVEN86A, KBF1, MBP1, Transfac |
| TGGnnnnnnKCCAR | N27, Xie X, et al. |
| TGTGGWWW | AP3, Transfac |
| TGTTTGY | HNF-3, Xie X, et al. |
| TGTYnnnnnRGCARM | N117, Xie X, et al. |
| TKNNGNAAK | NFIL6, Transfac |
| TKNNGYAAK | CEBP_03, Transfac |
| TMTCGCGAnR | N8, Xie X, et al. |
| TTAAAATTCA | OCT4*, Transfac |
| TTAACAGTCACCCCCAAC | MTTF1, Transfac |
| TTACGTAA | CREBP1_01, Transfac |
| TTAYGTAAY | E4BP4, Jaspar |
| TTAYRTAA | E4BP4, Xie X, et al. |
| TTAnTCA | AP-1(*), Xie X, et al. |
| TTAnWnAnTGGM | N161, Xie X, et al. |
| TTCACGCWTSANTK | Pax6, Jaspar |
| TTCATATTACTCT | GAF, Transfac |
| TTCYRGAA | N72, Xie X, et al. |
| TTCYnRGAA | STAT5A, Xie X, et al. |
| TTCnRGnnnnTTC | HSF, Xie X, et al. |
| TTGCWCAAY | C/EBPBETA, Xie X, et al. |
| TTGTTT | FOXO4, Xie X, et al. |
| TTTCATATTACTCT | AAF, Transfac |
| TTTSGCGC | E2F, Jaspar |
| TTTTSSCGS | E2F_01, Transfac |
| TTTnnAnAGCYR | N169, Xie X, et al. |
| TnCATnTCCYR | STAT1(*), Xie X, et al. |
| WAACAAT | SRY, Jaspar |
| WAWNTAGGTCA | RORalfa-2, Jaspar |
| WCAAnnnYCAG | N152, Xie X, et al. |
| WCTCnATGGY | N59, Xie X, et al. |
| WGATAR | GATA1, GATA2, GATA3, _01, Transfac |
| WGGAATGY | TEF-1, Xie X, et al. |
| WGMGGAA | SPI-B, Jaspar |
| WGRGGTCAAAGGTCA | PPARgamma-RXRal, Jaspar |
| WGTTnnnnnAAA | N155, Xie X, et al. |
| WNNANATA | FREAC-7, Jaspar |
| WNNANATAAAYA | FREAC7_01, Transfac |
| WTATYCAT | GHF1, Transfac |
| WTGAAAT | N174, Xie X, et al. |
| WTTGKCTG | N46, Xie X, et al. |
| WTTGYGGTY | AML-1, Jaspar |
| WWTAAGGC | N127, Xie X, et al. |
| WYAAAnnRnnnGCG | N126, Xie X, et al. |
| YAACKG | MYB_01, Transfac |
| YAATnAnRnnnCAG | N147, Xie X, et al. |
| YAATnRnnnYnATT | CART-1(*), Xie X, et al. |
| YACATTCCWSNG | TEF-1, Jaspar |
| YATGnWAAT | OCT-X, Xie X, et al. |
| YATTnATC | CDP(*), Xie X, et al. |
| YCAGCTGYGG | AP4, Transfac |
| YCATTAA | IPF1(*), Xie X, et al. |
| YGACnnYACAR | N149, Xie X, et al. |
| YGCAnTGCR | N96, Xie X, et al. |
| YGCGCAYGCGCR | NRF1, Transfac |
| YGCGYRCGC | N30, Xie X, et al. |
| YGGMNNNNNGCCAA | NF1_01, Transfac |
| YGGMNNNNNNGCCAA | NF1_01, Transfac |
| YGTCAGC | NFS, Transfac |
| YGTCCTTGR | N109, Xie X, et al. |
| YKACATTT | N145, Xie X, et al. |
| YRCCAKnnGnCGC | N156, Xie X, et al. |
| YRTCAnnRCGC | N115, Xie X, et al. |
| YTAATTAA | LHX3, Xie X, et al. |
| YTAAYnGCT | N168, Xie X, et al. |
| YTATTTTnR | MEF-2, Xie X, et al. |
| YTCCCRnnAGGY | N114, Xie X, et al. |
| YTTCCnnnGGAMR | N150, Xie X, et al. |
| YWATTWnnRGCT | N173, Xie X, et al. |
| YYCATTCAWW | POU1F1(*), Xie X, et al. |
| YnGTTnnnATT | N170, Xie X, et al. |
| YnTTTnnnAnGCARM | N166, Xie X, et al. |
| mrMMGGAWry | ELK1_01, Transfac |
| rckcTAWWWWTAgrwy | RSRF4_01, Transfac |
| GAGGTTGCAGTGA | CHOP-Ext, Thomo-LaTrobe |
| GRTTGCA | CHOP, Tomohisa-LaTrobe |
| AGAATNGCTNG | MURE1ext, Tomohisa-LaTrobe |
| AGAATBGCT | MURE1, Tomohisa-LaTrobe |
| AAAAAAAAAAAAAAAAAAAA | MultiA-JonA, LaTrobe |
| GYACBCSAG | MURE2, Tomohisa-LaTrobe |
| ATCTGGTT | MT3, Susuki et al |
| TGGTGTTAG | MT4, Susuki et al |
| SCACG | ATF6, Yoshida et al. |
| CCAATnnnnnnnnnCCACG | ERSE, Yoshida et al. |
